# A Retrospective Comparative Analysis of Outcomes and Prognostic Factors in Adult and Pediatric Patients with Osteosarcoma

**DOI:** 10.3390/curroncol28060443

**Published:** 2021-12-12

**Authors:** Stefano Testa, Benjamin D. Hu, Natalie L. Saadeh, Allison Pribnow, Sheri L. Spunt, Gregory W. Charville, Nam Q. Bui, Kristen N. Ganjoo

**Affiliations:** 1 Department of Medicine, Stanford University, Stanford, CA 94304, USA; 2 Department of Pediatrics, School of Medicine, Stanford University, Stanford, CA 94304, USA; benhu8@stanford.edu (B.D.H.); apribnow@stanford.edu (A.P.); sspunt@stanford.edu (S.L.S.); 3 Division of Oncology, Department of Medicine, Stanford University, Stanford, CA 94304, USA; saadeh@chapman.edu (N.L.S.); nambui@stanford.edu (N.Q.B.); 4 Department of Pathology, Stanford University, Stanford, CA 94304, USA; gwc@stanford.edu

**Keywords:** bone osteosarcoma, adult, pediatric, methotrexate, tumor necrosis

## Abstract

Osteosarcoma is the most common primary bone malignancy in both children and adults. Despite introduction of intensive multimodal treatment with chemotherapy and surgery, outcomes are still poor, especially for patients with metastatic disease and adults. Hence, there is an ongoing need for better prognostic markers and outcome data to inform management decisions in both the adult and pediatric setting. Here, we retrospectively analyzed 112 patients with bone osteosarcoma treated at two large adult and pediatric tertiary academic centers between 1989 and 2019. Patients were divided into an adult (≥18 years) and pediatric (<18 years) cohort for comparison. Our aim was to evaluate predictors of outcomes in pediatric and adult patients, with a specific focus on the role of methotrexate when added to a combination of doxorubicin-cisplatin; the prognostic value of tumor necrosis after neoadjuvant chemotherapy; and outlining any differences in outcomes between adults and pediatric patients that could inform clinical management. Adult patients treated with methotrexate-doxorubicin-cisplatin and those treated with doxorubicin-cisplatin had similar 5-year PFS (26%, 95%CI: 45.5%–10% vs. 50%, 95%CI: 69.6%–26.2%, *p* = 0.1) and 5-year OS (63%, 95%CI: 82%–34%, vs. 78%, 95%CI: 90.6%–52.6%, *p* = 0.5). In the adult cohort, there was no difference between patients with ≥90% necrosis and <90% necrosis in either 5-year PFS (42%, 95%CI: 71.1%–11.3% vs. 38%, 95%CI: 57.7%–18.2%, *p* = 0.4) or 5-year OS (85%, 95%CI: 97.8%–33.4% vs. 56%, 95%CI: 76.8%–27.6%, *p* = 0.4). In the pediatric cohort, compared to patients with <90% necrosis, those with ≥90% necrosis had significantly better 5-year PFS (30%, 95%CI: 49.3%–14.1% vs. 55%, 95%CI: 73.9%–38.5%, *p* = 0.003) and 5-year OS (64%, 95%CI: 80.8%–41.1% vs. 78%, 95%CI: 92%–60.9%, *p* = 0.04). Adult and pediatric patients had similar 5-year OS (69%, 95%CI: 83.2%–49.8% vs. 73%, 95%CI: 83.2%–59.3%, *p* = 0.8) and 5-year PFS (37%, 95%CI: 52.4%–22.9% vs. 43%, 95%CI: 56.2%–30.4% *p* = 0.3) even though the proportion of patients with ≥90% necrosis after neoadjuvant chemotherapy was higher for children compared to adults (60.3% vs. 30%, OR: 3.54, 95%CI: 1.38–8.46, *p* = 0.006). In conclusion, in adult patients, the addition of methotrexate to doxorubicin and cisplatin did not correlate with a significant survival benefit, questioning the therapeutic value of methotrexate overall. Our study confirms the prognostic utility of percent tumor necrosis after neoadjuvant chemotherapy in pediatric patients but not in adult patients. Lastly, this is one of the few reported studies where patients with osteosarcoma younger and older than 18 years had similar PFS and OS.

## 1. Introduction

Osteosarcoma is a primary bone cancer characterized by immature bone production. It is observed more frequently in males and has a bimodal age distribution with peaks of incidence in adolescents and individuals older than 60 years [1,2,3]. Annual incidence rates are approximately 3.8 per million in individuals aged ≤24 years, 1.6 per million in those 25–59 years, and 3.5 per million in patients ≥60 years [4]. Despite being a rare malignancy representing <1% of cancers in adults and 3–5% of childhood cancers, it is the most common primary bone tumor [5]. The prognosis of patients with high-grade osteosarcoma has dramatically improved during the last 50 years with the addition of systemic chemotherapy [6,7,8,9]. However, osteosarcoma still accounts for significant cancer morbidity and mortality in children and even more so in the elderly population, where prognosis is significantly worse [10,11,12,13]. In this study, we retrospectively analyzed pediatric and adult patients with osteosarcoma treated at two large adult and pediatric tertiary academic medical centers between 1989 and 2019. Our aim was to correlate outcomes with multiple patient and tumor-related variables in both adult and pediatric patients and to identify any differences between the two populations that could inform clinical management. Specifically, our aim was to answer several fundamental questions: (1) is there a difference in outcomes between patients treated with methotrexate-doxorubicin-cisplatin (MAP) and those treated with doxorubicin-cisplatin (AP); (2) what is the prognostic value of tumor necrosis after neoadjuvant chemotherapy in adult and pediatric patients; and (3) is there a difference in progression-free survival (PFS) and/or overall survival (OS) between adult and pediatric patients when they are treated in the same manner?

## 2. Materials and Methods

### 2.1. Recruitment

**Figure 1:**
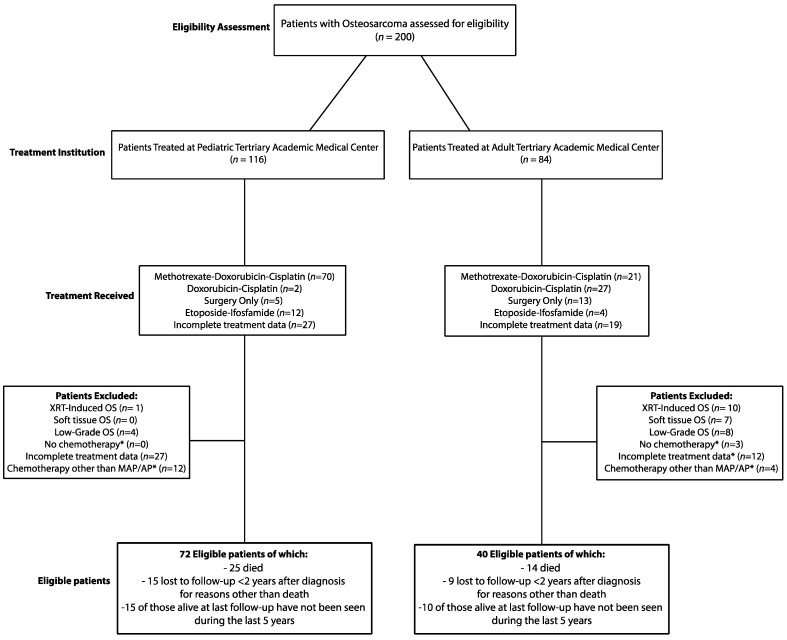
Patient Selection. (*) If not excluded for other criteria.

A total of 200 patients with pathologically confirmed high-grade bone osteosarcoma were assessed for eligibility (Figure 1). Of these, 116 were treated at a tertiary pediatric academic center, and 84 were treated at a tertiary adult academic center between 1989 and 2019. At both institutions, patients were treated with either surgery only or with MAP, AP or Etoposide-Ifosfamide (IE) chemotherapy, either in the adjuvant or neoadjuvant setting. Treatment data were incomplete for some patients at both centers since these patients received part of their care at other institutions. Patients with radiotherapy-induced osteosarcoma, extraosseous osteosarcoma, or low-grade osteosarcoma and patients who did not receive chemotherapy or were treated with any first-line chemotherapy regimen other than MAP or AP were excluded. Moreover, patients for whom there was incomplete treatment information were excluded from the study. Based on these criteria, a total of 40 patients were found to be eligible among those treated at the adult medical center, and of these, 14 died, 9 were lost to follow-up less than two years after the diagnosis for reasons other than death, and 10 of those alive at last follow-up had not been seen during the previous five years. Instead, of the patients treated at the pediatric center, 72 were found to be eligible, and among these, 25 died, 15 were lost to follow-up less than two years after the diagnosis for reasons other than death, and 15 of those *alive* at last follow-up had not been seen during the last five years. Eligible patients from both centers were then divided by age into an adult cohort (patients ≥ 18 years old, *n* = 45) and a pediatric cohort (patients < 18 years old, *n* = 67) for further comparative analysis.

### 2.2. Diagnostic Staging and Treatment

Standard baseline evaluations for patients with newly diagnosed osteosarcoma at our institutions include magnetic resonance imaging (MRI) of the primary tumor, computed tomography (CT) of the chest, and either a Tc99m bone scan or an fluorodeoxyglucose (FDG)-positron emission tomography (FDG-PET/CT or PET/MRI scan) of the whole body to assess for the presence of metastases. Surveillance follow-up tests for all patients included radiographs of the primary site and CT scan of the chest. Patients were treated with a combination of doxorubicin and cisplatin with or without methotrexate, according to published protocols. Specifically, doxorubicin was administered at a dose of 75 mg/m^2^ per cycle, cisplatin was administered at a dose of 120 mg/m^2^ per cycle, and methotrexate was administered at a dose of 12 g/m^2^ per cycle. Patients with axial osteosarcoma underwent total resection of the tumor whenever feasible with the aim of achieving negative margins. For extremity osteosarcoma, patients either underwent a limb-sparing tumor resection or an amputation. For patients who received neoadjuvant chemotherapy, surgery was performed 11 weeks after the start of chemotherapy whenever possible (range 11 to 16 weeks), and pathologic response was reported as percent necrosis in the resected tumor. Patients who received upfront surgery without neoadjuvant chemotherapy were excluded from the assessment of pathologic tumor response since percent tumor necrosis was not measured for these patients.

### 2.3. Statistical Analysis

Statistical analysis was performed using Python packages for statistics and GraphPad Prism. A Student’s *t*-test was used to detect differences in continuous variables, including percent necrosis after neoadjuvant chemotherapy and age at diagnosis. The chi-square test was used to measure differences in categorical variables between groups. The log-rank test was used to test for survival differences. Survival curves were constructed using the Kaplan–Meier method. OS was calculated from the time of diagnosis to the time of death or censored at time of last follow up. PFS was calculated from the time of diagnosis to the time of the first detected local or distant recurrence. *p*-Values lower than 0.05 were considered statistically significant.

## 3. Results

### 3.1. Clinical Features and Treatment

Patients in the adult cohort had a median age of 32 years and range 18–71, and patients in the pediatric cohort had a median age of 14 and range 7–17 (Table 1).

**Figure 2:**
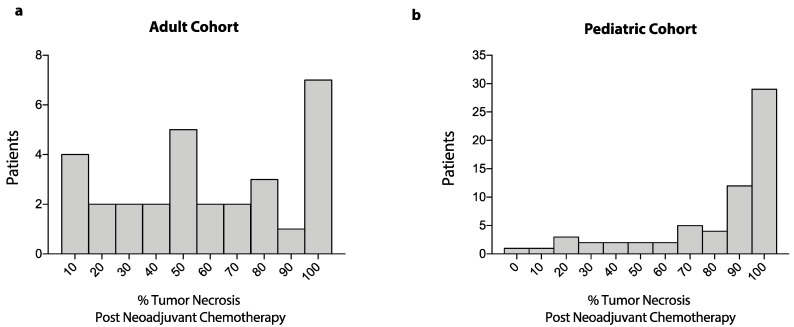
Frequency distribution of tumor necrosis after neoadjuvant chemotherapy. Data show frequency distribution of percent tumor necrosis after neoadjuvant chemotherapy for patients in the adult cohort (**a**) and patients in the pediatric cohort (**b**).

There were no significant differences in sex or presence of primary metastatic disease between adult and pediatric patients. Axial tumors were significantly more frequent in the adult compared to the pediatric population (28.9% vs. 5.9%), while extremity tumors were more frequent in the pediatric compared to the adult cohort (94.1% vs. 71.1%, OR: 0.15, 95%CI: 0.05–0.47, *p* = 0.0009). For extremity osteosarcoma, limb-sparing surgery was performed more frequently in adult compared to pediatric patients (90.6% vs. 73%), whereas limb amputations were performed more often in the pediatric cohort (26.9% vs. 9.4%, OR: 0.27, 95%CI: 0.08–0.93, *p* = 0.04). Among patients with axial osteosarcoma, in the adult cohort, all 13 patients (100%) underwent resection, while in the pediatric cohort, three of four patients (75%) underwent axial tumor resection (OR: 0.0, 95%CI: 0.0–2.76, *p* = 0.06). Regarding chemotherapy, in the adult cohort, 51.1% of patients received AP, and 48.9% received MAP; in the pediatric cohort, 97% of patients received MAP, and only 3% were treated with AP (OR: 33.9, 95%CI: 8.43–150.2, *p* < 0.0001). In the pediatric cohort, the proportion of patients with ≥90% necrosis after neoadjuvant chemotherapy was significantly higher compared to the adult cohort (60.3% vs. 30%, OR: 3.54, 95%CI: 1.38–8.46, *p* = 0.006). Similarly, both the mean and median tumor necrosis following neoadjuvant chemotherapy were higher in the pediatric cohort compared to the adult cohort (Figure 2). Additionally, mean tumor necrosis was higher in pediatric patients that received neoadjuvant MAP compared to adult patients that received neoadjuvant MAP (78.4% ± SD 27.4% vs. 57.4% ± SD: 32.6%, MD: 21.0%, 95%CI: 36.6%–5.4%, *p* = 0.009).

### 3.2. Prognostic Value of Tumor Necrosis after Neoadjuvant Chemotherapy

**Figure 3:**
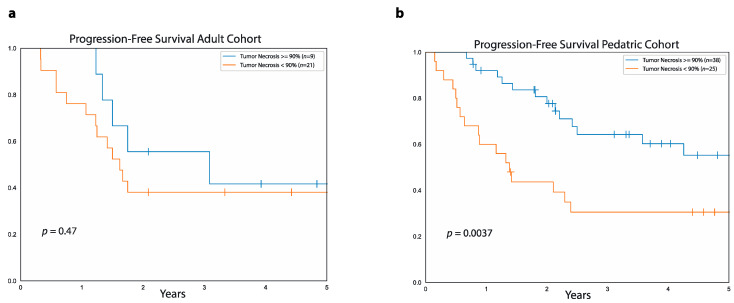
Relationship between PFS and tumor necrosis after neoadjuvant chemotherapy. Data show progression-free survival for patients with ≥90% tumor necrosis (blue curve) and <90% necrosis (orange curve) after neoadjuvant chemotherapy in the adult cohort (**a**) and the pediatric cohort (**b**).

In the adult cohort, five-year PFS was similar between patients with good pathological response, defined as ≥90% tumor necrosis after neoadjuvant chemotherapy (42%, 95%CI: 71.1%–11.3%), and those with poor pathological response, defined as <90% necrosis (38%, 95%CI: 57.7%–18.2%, *p* = 0.4, Figure 3). Moreover, there was no difference in five-year OS between adult patients with good pathological response (85%, 95%CI: 97.8%–33.4%) and those with poor pathological response (56%, 95%CI: 76.8%–27.6%, *p* = 0.4).

In contrast, in the pediatric cohort, five-year PFS was higher in patients with ≥90% necrosis (55%, 95%CI: 73.9%–38.5%) compared to patients with <90% tumor necrosis (30%, 95%CI: 49.3%–14.1%, *p* = 0.003). Five-year OS was also higher for good responders (78%, 95%CI: 92%–60.9%) compared to poor responders (64%, 95%CI: 80.8%–41.1% *p* = 0.04) in the pediatric cohort.

To explain the lack of correlation between pathologic tumor response and outcomes in the adult cohort, we stratified patients by age at diagnosis, type of chemotherapy received, site of primary tumor, and presence or absence of metastases at diagnosis (Tables S1 and S2). In this case, again, we found no difference in either PFS or OS between patients with <90% and ≥90% necrosis in each stratum analyzed.

In the adult cohort, median follow-up was 4.7 years (range: 1.9 to 6 years) for the seven surviving patients with good pathological response and 3.7 years (range: 1 to 9.2 years) for the 14 surviving patients with poor pathological response. Instead, in the pediatric cohort, median follow-up was 4.3 years (range: 9 months to 14.6 years) for patients with good pathological response and 4.7 years (range: 1.4 to 11 years) for patients with poor pathological response.

### 3.3. Differences in Outcome Based on Chemotherapy Regimen

**Figure 4:**
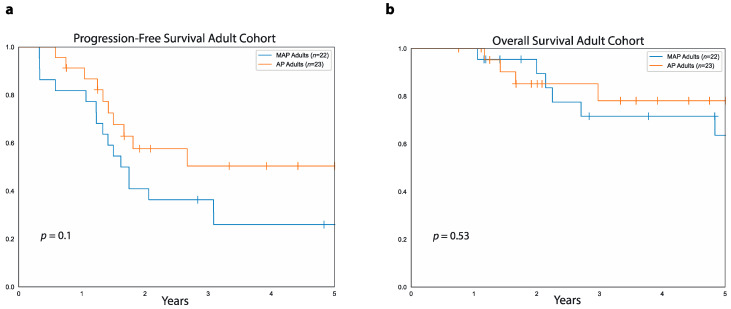
MAP vs. AP in adult patients with osteosarcoma. (**a**) Progression-free survival in the adult cohort for patients treated with MAP (blue curve) versus patients treated with AP (orange curve). (**b**) Overall survival for patients treated with MAP (blue curve) and patients treated with AP (orange curve) in the adult cohort.

In the pediatric cohort, 65 patients were treated with MAP, and only two were treated with AP, making it difficult to compare the two groups. In contrast, in the adult cohort, 22 patients were treated with MAP and 23 with AP either in the adjuvant or neoadjuvant setting (Figure 4). In this cohort, the five-year PFS was similar between patients treated with AP (50%, 95%CI: 69.6%–26.2%) and those treated with MAP (26%, 95%CI: 45.5%–10%, *p* = 0.1). As far as five-year OS, this was also similar between adult patients treated with AP (78%, 95%CI: 90.6%–52.6%) and those treated with MAP (63%, 95%CI: 82%–34%, *p* = 0.5).

In the adult cohort, 31.8% of patients treated with MAP had primary metastases versus 21.7% of those treated with AP (OR: 0.59, 95%CI: 0.16–2.4, *p* = 0.4, Table 2). In adult patients with primary metastases, we found no difference between patients treated with MAP and those treated with AP either in terms of five-year PFS (14%, 95%CI: 46.5%–0.8%, vs. 0%, 95%CI: 0%–0%, *p* = 0.7) or in terms of five-year OS (67%, 95%CI: 90.7%–20% vs. 0%, 95%CI: 0%–0%, *p* = 0.1). Similarly, among adult patients with localized disease at diagnosis, we found no difference between those treated with MAP and those treated with AP, both in terms of five-year PFS (32%, 95%CI: 55.7%–10.9% vs. 58%, 95%CI: 77.7%–30.6%, *p* = 0.1) and five-year OS (60%, 95%CI: 83.8%–22.1% vs. 89%, 95%CI: 97.2% vs. 61.5%, *p* = 0.2).

Furthermore, patients treated with MAP and AP in the adult cohort were similar in terms of sex, primary tumor location, type of surgery received regardless of the site of the primary tumor, and pathological tumor response. In particular, 35.3% of the adult patients who received neoadjuvant MAP achieved a good pathological response compared to 23.1% of the patients who received neoadjuvant AP (OR: 1.18, 95%CI: 0.36–7.88, *p* = 0.4). In addition, adult patients treated with MAP were significantly younger than those treated with AP (mean age in years: 27.3 ±: SD 7.8 vs. 40.7 ±: SD 15.1, MD: 13.5, 95%CI: 6.2–20.8, *p* = 0.0006). Median follow-up was 4.4 years (range: 1 to 8.3 years) for the 15 surviving patients in the adult cohort treated with MAP and 3.6 years (range: 9 months to 9.2 years) for those 19 surviving adult patients treated with AP.

### 3.4. Differences in Outcome Based on Presence of Primary Metastases

**Figure 5:**
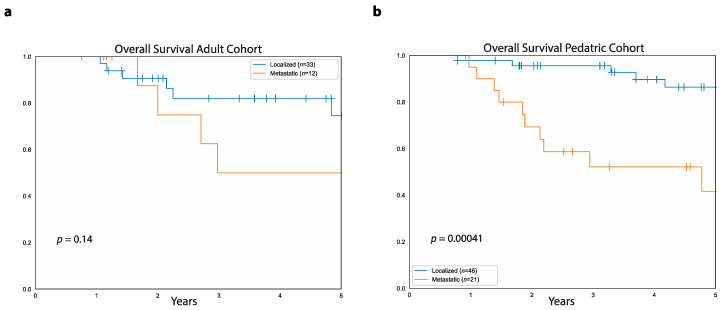
Relationship between OS and metastatic disease at diagnosis. (**a**) Data shows overall survival in the adult cohort for patients with absence of primary metastases (blue curve) and patients with primary metastatic disease (orange curve). (**b**) Overall survival in the pediatric cohort for patients without primary metastatic disease (blue curve) and patients with primary metastases (orange curve).

Metastatic disease at presentation correlated with poor outcomes in both the adult and pediatric cohorts. In both cohorts combined, five-year PFS was higher in patients with localized disease at diagnosis compared to those with primary metastatic disease (54.6%, 95%CI: 65.7%–41.8% vs. 7.2%, 95%CI: 20.5%–1.3%, *p* < 0.0001). Similarly, five-year OS was higher in patients with localized disease at diagnosis compared to those with primary metastatic disease (82.3%, 95%CI: 90%–69.7% vs. 46.3%, 95%CI: 64.5%–25.8%, *p* = 0.0002). In the adult cohort, five-year PFS was higher in patients with localized disease at diagnosis compared to those with primary metastases (46.4%, 95%CI: 62.8%–28.2% vs. 10.8%, 95%CI: 37.7%–0.7%, *p* = 0.02), while there was no difference in five-year OS between patients with primary localized disease (75%, 95%CI: 88.5%–50.8%) and those with primary metastatic disease (50%, 95%CI: 77.3%–14.9%, *p* = 0.1, Figure 5a).

In the pediatric cohort, five-year PFS was higher in patients with primary localized disease compared to those with primary metastatic disease (60.9%, 95%CI: 74.7%–43% vs. 5.2%, 95%CI: 21.3%–0.4%, *p* < 0.0001). Similarly, pediatric patients with primary localized disease had higher five-year OS (86%, 95%CI: 93.8%–69.5%) compared to those with primary metastases (42%, 95%CI: 65.1%–18.8%, *p* = 0.0004, Figure 5b).

Considering only patients with primary metastases, there was no difference between patients in the adult cohort and those in the pediatric cohort both in terms of five-year PFS (10.8%, 95%CI: 37.7%–0.7% vs. 5.2, 95%CI: 21.3%–0.4%, *p* = 0.2) and five-year OS (50.8%, 95%CI: 78.1%–15.7% vs. 43.5, 95%CI: 65.6%–19.3%, *p* = 0.7). Similarly, in patients with localized disease, there was no difference between adult and pediatric patients both in regards to five-year PFS (46.4%, 95%CI: 62.8%–28.2% vs. 60.9, 95%CI: 74.7%–43%, *p* = 0.07) and five-year OS (75.6%, 95%CI: 89.1%–50.7% vs. 86.3, 95%CI: 94.1%–69.8%, *p* = 0.5).

Median follow-up in the adult cohort was 3.7 years (range: 1.2 to 9.2 years) for the 27 surviving patients with localized disease at diagnosis and 1.9 years (range: 9 months to 8.3 years) for the seven surviving patients with primary metastases. Instead, in the pediatric cohort, median follow-up was 4.7 years (range: 9 months to 14.6 years) for the 36 surviving patients with localized disease and 4.1 years (range: 11 months to 9 years) for the 10 surviving patients with primary metastases.

### 3.5. Survival Differences between Adult and Pediatric Cohort

**Figure 6:**
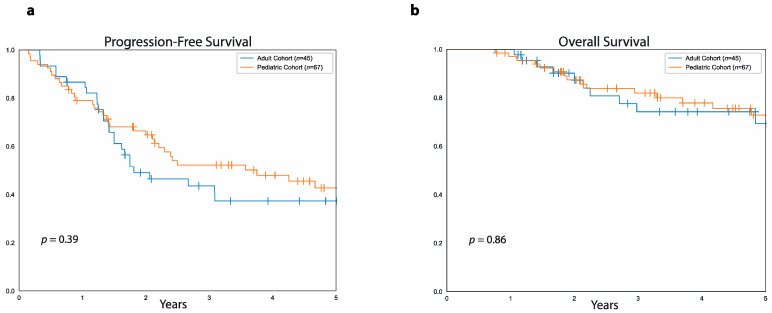
PFS and OS in the adult versus the pediatric cohort. (**a**) Progression-free survival in patients of the adult cohort (blue curve) and patients of the pediatric cohort (orange curve). (**b**) Overall survival in patients of the adult cohort (blue curve) and patients of the pediatric curve (orange curve).

There was no difference between the adult and pediatric cohorts as a whole either in five-year PFS (37%, 95%CI: 52.4%–22.9% vs. 43%, 95%CI: 56.2%–30.4% *p* = 0.3) or five-year OS (69%, 95%CI: 83.2%–49.8% vs. 73%, 95%CI: 83.2%–59.3%, *p* = 0.8, Figure 6). Median follow-up was 3.7 years (range: 9 months to 9.2 years) for the 34 surviving patients in the adult cohort and 4.6 years (range: 9 months to 14.6 years) for the 46 survivors in the pediatric cohort.

**Figure 7:**
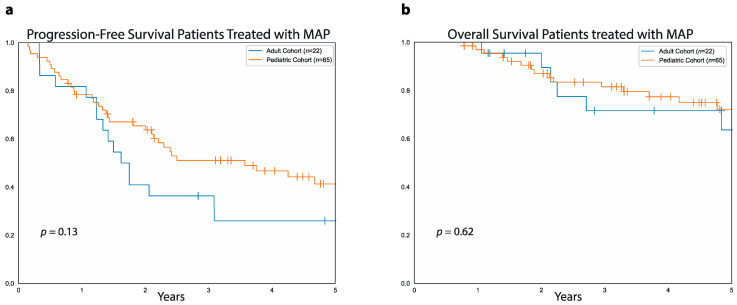
PFS and OS in pediatric and adult patients treated with MAP. (**a**) Data show progression-free survival for patients treated with MAP in the adult cohort (blue curve) and patients treated with MAP in the pediatric cohort (orange curve). (**b**) Overall survival for pediatric patients (orange curve) and adult patients (blue curve) treated with MAP.

We then compared PFS and OS between adult and pediatric patients who received MAP, considering that most of the patients in the pediatric cohort were treated with MAP. In this case, there was no difference between adult and pediatric patients that received MAP either in terms of five-year PFS (26%, 95%CI: 45.5%–10% vs. 41%, 95%CI: 53.6%–27.4% *p* = 0.1, Figure 7a) or in terms of five-year OS (64%, 95%CI: 83%–35% vs. 72%, 95%CI: 81%–58%, *p* = 0.6, Figure 7b). Of note, the proportion of patients with metastatic disease at diagnosis was similar between patients in the adult cohort treated with MAP and those in the pediatric cohort treated with MAP (36.4% vs. 32.3%, OR: 1.19, 95%CI: 0.41–3.35, *p* = 0.7). In surviving patients who received MAP, median follow-up was 4.4 years (range: 1 to 8.3 years) in the adult cohort and 4.6 years (range: 9 months to 14.6 years) in the pediatric cohort.

## 4. Discussion

Osteosarcoma is the most frequent primary bone cancer in both children and adults, and despite introduction of multimodal treatment with surgery and chemoradiotherapy, outcomes are still poor for a significant number of patients. In this study, we retrospectively correlated multiple variables with progression-free and overall survival in both adult (≥18 years old) and pediatric (<18 years old) patients with high-grade osteosarcoma. The main aim of this study was to assess the strength and value of different variables as prognostic factors in patients with osteosarcoma to find any differences between the adult and pediatric populations that could inform clinical decision making. In particular, we focused on the therapeutic role of methotrexate when added to a combination of doxorubicin-cisplatin; the prognostic value of pathologic tumor necrosis after neoadjuvant chemotherapy; and outlining any differences in OS or PFS between adult and pediatric patients when they are treated similarly. As far as the role of methotrexate, we were only able to meaningfully assess this in the adult population since only 2/67 patients in the pediatric cohort received AP rather than MAP. Our results show similar OS and PFS for adult patients treated with MAP compared to AP, thus questioning the value of methotrexate in this setting. Regarding the prognostic strength of pathological tumor necrosis after neoadjuvant chemotherapy, we found that tumor necrosis ≥90% positively correlated with higher PFS and OS in the pediatric cohort but not in the adult cohort, questioning the value of tumor necrosis as a prognostic factor in patients older than 18 years. Lastly, we found no difference in either PFS or OS between the adult and pediatric cohort, either when considered as a whole or when looking only at patients treated with MAP in each cohort.

However, this study has several limitations that must be considered when interpreting these results. First, the small sample size of the cohorts, related to the low incidence of osteosarcoma, with consequent lack of statistical power, makes our observations prone to type II error. In addition, data were obtained from patients treated over 30 years, with associated changes in standard of care over time and increase in heterogeneity within the cohorts due to differences between patients treated at different points in time. Moreover, a considerable number of patients in both cohorts were either lost to follow-up less than two years after diagnosis or had not been seen during the five years preceding this analysis, which can affect the precision of our observations, especially regarding overall survival.

As far as the optimal chemotherapy for adults with osteosarcoma, this is not established given the scarcity of randomized data in this setting. Most of the chemotherapy regimens used in adults are based on clinical trials conducted in patients younger than 40 years of age [6]. The most used chemotherapy combination in adults is AP with addition of high-dose methotrexate for younger patients with good performance status. In our adult population, there was no difference in five-year PFS or OS in patients treated with AP compared to MAP even though the latter were younger than the former. Overall, these findings raise the question of whether giving methotrexate is worthwhile even in younger adults. Bramwell and colleagues reported a better five-year disease-free survival (DFS) in patients younger than 40 treated with AP compared to MAP (57% vs. 41% *p* = 0.02). However, in this study, the five-year OS was similar between patients older and younger than 40 years (64% versus 50%, *p* = 0.1) [14]. Of note, the populations in this study were significantly younger than our adult cohort, with a median age of 16 (range 3 to 40 years) for patients treated with AP and a median age of 15 (range 4 to 34 years) for patients treated with MAP. In addition, the patients in the AP arm of this study received two additional cycles of chemotherapy compared to those in the MAP arm. A different prospective study in the pediatric setting also showed that methotrexate could be safely eliminated without compromising five-year event-free or OS although this was in the context of a regimen composed of Ifosfamide, carboplatin, and doxorubicin [15]. Therefore, these studies together with our own suggest that the addition of methotrexate to doxorubicin and cisplatin for treatment of adult patients with osteosarcoma may not provide significant disease control and survival advantage. Eliminating high-dose methotrexate may also improve therapy tolerance and quality of life in the adult population if we consider the increased duration of treatment related to the delayed clearance of methotrexate in adults compared to pediatric patients [16]. However, larger multi-centric randomized prospective studies will be needed to better determine whether methotrexate provides any benefit in adult patients. Similar to the adult population, the contribution of high-dose methotrexate to the treatment of pediatric patients with osteosarcoma has not been adequately studied in randomized trials even though the benefits of multi-agent chemotherapy in terms of both PFS and OS are well documented [17,18]. In our study, 95.5% of the patients in the pediatric cohort were treated with MAP either as neoadjuvant or adjuvant treatment, and pediatric patients generally tolerate high-dose methotrexate better than their adult counterparts. The outcomes of our pediatric cohort are similar to what has been previously reported for pediatric patients with osteosarcoma treated with MAP-based chemotherapy [19,20,21]. Given the emerging evidence that high-dose methotrexate may not add significantly to the efficacy of AP, future studies in pediatric patients could evaluate eliminating methotrexate in favor of other promising novel agents.

We also assessed the prognostic value of the extent of tumor necrosis after neoadjuvant chemotherapy in our adult and pediatric cohorts. In general, patients who achieve ≥90% tumor necrosis have better outcomes compared to those with <90% necrosis, and most of the data to support this come from studies in the pediatric population [22,23,24]. Of the few studies available in the adult setting, a randomized controlled trial from Patel et al., which enrolled a total of 19 patients, showed higher five-year OS in patients with more than 90% necrosis after neoadjuvant chemotherapy versus those with less than 90% necrosis (100% vs. 50%, *p* = 0.02) [25]. Moreover, data from 37 adult patients with osteosarcoma treated at the MD Anderson Cancer Center between 1980 and 1991 demonstrated higher five-year DFS for patients with good compared to poor pathological response to neoadjuvant chemotherapy (80% vs. 10%, *p* < 0.05) [26]. Instead, in the pediatric setting, there is ample evidence of a positive correlation between pathological tumor response to neoadjuvant chemotherapy and outcomes [21]. For example, a retrospective analysis from the Cooperative Osteosarcoma Study Group showed five-year OS of 77.8% and five-year EFS of 67.6% for 734 patients with ≥90% tumor necrosis after neoadjuvant chemotherapy versus, respectively, 55.5% and 38.6% for 586 patients with <90% necrosis, including in the analysis both patients with metastatic and localized disease at diagnosis [27]. Similarly, a different study that evaluated a total of 1058 patients showed higher five-year OS in good responders compared to poor responders but only among the 911 patients with localized disease at diagnosis (76.1% vs. 56.1%, *p* = 0.0001), while no differences in survival were observed between good responders and poor responders among patients with metastatic disease at presentation [24]. In accordance with these data, in our pediatric cohort, a good response to neoadjuvant chemotherapy, defined as ≥90% tumor necrosis in the resected tumor, strongly correlated with higher five-year PFS and OS. In contrast, in our adult cohort, there was no difference in either five-year PFS or five-year OS between patients who achieved a good pathological response to neoadjuvant chemotherapy and those who did not. Interestingly, when performing a stratified analysis of PFS by age at diagnosis in the adult cohort, there was a trend for better PFS in patients with ≥90% necrosis among those younger than 32 years, which barely failed to meet statistical significance (*p* = 0.06). We also observed lower rates of good pathologic response in the adult cohort compared to the pediatric cohort similar to prior studies where older patients were less likely to achieve a good pathologic response compared to younger patients [27]. Overall, our results together with previous data suggests that there might be age-related differences in tumor necrosis after neoadjuvant chemotherapy, with younger patients being more likely to achieve a good pathologic response, correlating with better outcomes. This age-related variability might be linked to differences in the biology and pathogenesis of osteosarcoma between adult and pediatric patients. However, based on our data, it is not possible to draw definitive conclusions regarding the prognostic role of tumor necrosis in patients older than 18 years, both due to the small size of our sample and to the high percentage of censored cases.

Lastly, in our study, adult and pediatric patients had very similar outcomes. There was indeed no difference in five-year PFS and OS between the adult and pediatric populations either as a whole or considering only patients that received MAP in each cohort. In addition, PFS and OS were similar between adult and pediatric patients when evaluating those with localized and metastatic disease separately in each cohort. Overall, these results are in contrast with available data, which suggest that younger patients tend to fare better than older patients [1]. Prior larger prospective studies have shown that patients over 18 years have a significantly poorer EFS and OS than younger patients due to an increased rate of tumor recurrence [13]. Our findings might be at least in part explained by the age distribution of the cohorts of our study given that all the patients in the pediatric cohort and 80% of the patients in the adult cohort were younger than 45 years. In addition, in our study, five-year OS rates were higher than what has been previously shown for both the adult and pediatric populations. These differences might be explained by the fact that care quality indicators such as preoperative imaging, adequate surgical margins, and chemotherapy administration have been reported to be reached more often in patients treated at sarcoma centers [28].

## 5. Conclusions

In summary, in this study adding methotrexate to a combination of doxorubicin and cisplatin did not correlate with better outcomes for adult patients with osteosarcoma, questioning the benefit of methotrexate-based chemotherapy in young adults. Moreover, in the adult cohort of this study, higher tumor necrosis after neoadjuvant chemotherapy did not necessarily correlate with improved outcomes, thus challenging the validity of the extent of tumor necrosis at resection as a prognostic factor in patients with osteosarcoma older than 18 years. Lastly, this represents one of the few available retrospective studies where PFS and OS are similar between adult and pediatric patients with osteosarcoma. However, definitive answers to the questions we posed cannot be obtained based on our data, and larger, prospective, multi-centric studies will be needed to better evaluate the relationship between pathologic necrosis and outcome and to evaluate if methotrexate can be eliminated from the treatment of osteosarcoma without compromising progression-free or overall survival.

## Figures and Tables

**Table 1:** Adult cohort vs. pediatric cohort.

	Adult Cohort	Pediatric Cohort	OR/MD (95%CI)	*p*-Value
**Age**				
Mean ± SD, years	34.1 ± 13.7	13.4 ± 3.0	20.7 (17.2–24.1)	**<0.0001** *
Median, years	32	14		
Range, years	(18–71)	(7–17)		
**Sex**				
Male, N (%)	27/45 (60%)	42/67 (62.7%)	1.12 (0.50–2.44)	0.774 †
Female, N (%)	18/45 (40%)	25/67 (37.3%)		
**Primary metastases**				
Absent, N (%)	33/45 (73.3%)	46/67 (68.7%)	1.25 (0.56–2.77)	0.594 †
Detected, N (%)	12/45 (26.7%)	21/67 (31.3%)		
**Site**				
Axial, N (%)	13/45 (28.9%)	4/67 (5.9%)	0.15 (0.05–0.47)	**0.0009** †
Extremities, N (%)	32/45 (71.1%)	63/67 (94.1%)		
**Chemotherapy**				
Adjuvant MAP, N (%)	4/45 (8.9%)	1/67 (1.5%)	33.9 (8.43–150.2)	**<0.0001** †
Neoadjuvant MAP, N (%)	18/45 (40%)	64/67 (95.5%)		
Adjuvant AP, N (%)	9/45 (20%)	0/67 (0%)		
Neoadjuvant AP, N (%)	14/45 (31.1%)	2/67 (3.0%)		
**Tumor Necrosis**				
Mean ± SD, %	57.6 ± 31.5	78.8 ± 27.1	21.3 (8.7–33.9)	**0.0011** *
Median, %	55	90		
Range, %	(10–100)	(0–100)		
≥90% Necrosis, N (%)	9/30 (30%)	38/63 (60.3%)	3.54 (1.38–8.46)	**0.006** †
<90% Necrosis, N (%)	21/30 (70%)	25/63 (39.7%)		
**Surgery**				
Extremities				
Limb-Sparing, N (%)	29/32 (90.6%)	46/63 (73%)	0.27 (0.08–0.93)	**0.046** †
Amputation, N (%)	3/32 (9.4%)	17/63 (26.9%)		
Axial				
Resection, N (%)	13/13 (100%)	3/4 (75%)	0.0 (0.0–2.76)	0.063 †
No, N (%)	0/0 (0%)	1/4 (25%)		

(*) *t*-test; (†) chi-square test; OR, Odds Ratio; MD, Mean Difference; 95%CI, 95% Confidence Interval; AP, Doxorubicin-Cisplatin; MAP, Methotrexate-Doxorubicin-Cisplatin; SD, standard deviation.

**Table 2:** MAP vs. AP chemotherapy in the adult cohort.

	MAP	AP	OR/MD (95%CI)	*p*-Value
**Age**				
Mean ± SD, years	27.3 ± 7.8	40.7 ± 15.1	13.5 (6.2–20.8)	**0.0006** *
Median, years	26	40		
Range, years	(18–48)	(21–71)		
**Sex**				
Male, N (%)	13/22 (59%)	14/23 (60.9%)	0.92 (0.30–2.85)	0.903 ^†^
Female, N (%)	9/22 (41%)	9/23 (39.1%)		
**Primary metastases**				
Absent, N (%)	15/22 (68.2%)	18/23 (78.3%)	0.59 (0.16–2.40)	0.444 ^†^
Detected, N (%)	7/22 (31.8%)	5/23 (21.7%)		
**Site**				
Axial, N (%)	5/22 (22.7%)	8/23 (34.8%)	1.81 (0.48–6.46)	0.372 ^†^
Extremities, N (%)	17/22 (77.3%)	15/23 (65.2%)		
**Tumor Necrosis**				
Mean ± SD, %	57.3 ± 32.6	64.4 ± 29.1	7.0 (30.5– -16.5)	0.545 *
Median, %	50	70		
Range, %	(10–100)	(10–98)		
≥90% Necrosis, N (%)	6/17 (35.3%)	3/13 (23.1%)	1.18 (0.36–7.88)	0.469 ^†^
<90% Necrosis, N (%)	11/17 (64.7%)	10/13 (76.9%)		
**Surgery**				
Extremities				
Limb-Sparing, N (%)	15/17 (88.2%)	14/15 (93.3%)	0.53 (0.03–5.09)	0.621 ^†^
Amputation, N (%)	2/17 (11.8%)	1/15 (6.7%)		
Axial				
Resection, N (%)	5/5 (100%)	8/8 (100%)		
No, N (%)	0/0 (0%)	0/0 (0%)		

(*) *t*-test; (†) chi-square test; OR, Odds Ratio; MD, Mean Difference; 95%CI, 95% Confidence Interval; AP, Doxorubicin-Cisplatin; MAP, Methotrexate-Doxorubicin-Cisplatin; SD, standard deviation.

## Data Availability

The data and supporting findings of this study are available from the corresponding author, S.T., upon reasonable request.

## Supplementary Materials

The following are available online at www.mdpi.com/article/10.3390/curroncol28060443/s1, Table S1: Stratified analysis of the relationship between OS and tumor necrosis in the adult cohort, Table S2: Stratified analysis of the relationship between PFS and tumor necrosis in the adult cohort.
