# Supplementary material for: A Retrospective Comparative Analysis of Outcomes and Prognostic Factors in Adult and Pediatric Patients with Osteosarcoma
Source: Curr Oncol. 2021 Dec 12;28(6):5304–17. doi: 10.3390/curroncol28060443 (PMC8700626; doi:10.3390/curroncol28060443)
Supplement: Supplementary file 1 [file curroncol-28-00443-s001.zip › curroncol-1435685-supplementary.pdf]

Table S1. Stratified analysis of the relationship between OS and tumor necrosis in the adult cohort.

		≥90% Necrosis	<90% Necrosis	
	**N (%)**	**5-year OS (95%CI)**	**5-year OS (95%CI)**	***p*-Value**
**Age**				
≥32 years	16/30 (53%)	100% (N/A)	69.3% (89.1%–31.2%)	0.297
<32 years	14/30 (47%)	66.7% (94.5%–5.4%)	44.4% (74.8%–10.3%)	0.299
**Chemotherapy**				
MAP	17/30 (57%)	75.0% (96.0%–12.8%)	54.0% (80.0%–18.2%)	0.439
AP	13/30 (43%)	100% (N/A)	66.7% (87.9%–28.2%)	0.218
**Primary metastases**				
Absent	23/30 (77%)	100% (N/A)	60.5% (82.4%–27.1%)	0.148
Detected	7/30 (23%)	0.0% (0.0%–0.0%)	50.0% (84.5%–5.8%)	0.916
**Site**				
Axial	6/30 (20%)	100% (N/A)	50.0% (91.0%–0.6%)	0.317
Extremities	24/30 (80%)	83.3% (97.5%–27.3%)	58.7% (77.6%–28.8%)	0.312

95%CI, 95% Confidence Interval; AP, Doxorubicin-Cisplatin; MAP, Methotrexate-Doxorubicin-Cisplatin.

Table S2. Stratified analysis of the relationship between PFS and tumor necrosis in the adult cohort.

		≥90% Necrosis	<90% Necrosis	
	**N (%)**	**5-year PFS (95%CI)**	**5-year PFS (95%CI)**	***p*-Value**
Age				
≥32 years	14/30 (47%)	25.0% (66.5%–0.9%)	50.0% (73.6%–20.9%)	0.488
<32 years	16/30 (53%)	75.0% (96.0%–12.8%)	20.0% (47.4%–3.1%)	0.064
Chemotherapy				
MAP	17/30 (57%)	50.0% (84.5%–5.8%)	23.1% (47.5%–5.6%)	0.289
AP	13/30 (43%)	50.0% (84.5%–5.8%)	55.6% (80.5%–20.5%)	0.970
Primary metastases				
Absent	23/30 (77%)	62.5% (89.3%–14.2%)	41.2% (62.7%–18.6%)	0.215
Detected	7/30 (23%)	25.0% (66.5%–0.9%)	20.0% (58.1%–0.8%)	0.688
Site				
Axial	6/30 (20%)	50.0% (91.0%–0.6%)	25.0% (66.5%–0.9%)	0.734
Extremities	24/30 (80%)	50.0% (80.4%–11.1%)	38.9% (59.9%–17.5%)	0.437

95%CI, 95% Confidence Interval; AP, Doxorubicin-Cisplatin; MAP, Methotrexate-Doxorubicin-Cisplatin.
